# Inflammatory-based prognostic indicators in prostate cancer: evaluating NLR, PLR, and SII in relation to Cambridge and ISUP classifications

**DOI:** 10.3389/fonc.2025.1595000

**Published:** 2025-07-04

**Authors:** Patryk Patrzałek, Anita Froń, Maja Mielczarek, Jakub Karwacki, Grzegorz Lesiuk, Dawid Janczak, Krystian Nagi, Wojciech Krajewski, Paweł Dębiński, Tomasz Szydełko, Bartosz Małkiewicz

**Affiliations:** ^1^ University Center of Excellence in Urology, Department of Minimally Invasive and Robotic Urology, Wroclaw Medical University, Wroclaw, Poland; ^2^ Division of Chemistry and Immunochemistry, Department of Biochemistry and Immunochemistry, Wroclaw Medical University, Wroclaw, Poland; ^3^ Regional Specialist Hospital, Wroclaw, Poland; ^4^ Department of Pathophysiology, Wroclaw Medical University, Wroclaw, Poland; ^5^ Department of Mechanics, Materials and Biomedical Engineering, Wroclaw University of Science and Technology, Wroclaw, Poland; ^6^ Clinical Urology Department, 4th Military Clinical Hospital, Wroclaw, Poland; ^7^ University Center of Excellence in Urology, Wroclaw Medical University, Wroclaw, Poland

**Keywords:** neutrophil-to-lymphocyte ratio, platelet-to-lymphocyte ratio, systemic immune-inflammation index, PCA, inflammatory markers, Cambridge prognostic groups

## Abstract

**Introduction:**

Systemic inflammation is increasingly recognized for its role in cancer prognosis. Inflammatory markers such as the neutrophil-to-lymphocyte ratio (NLR), platelet-to-lymphocyte ratio (PLR), and systemic inflammation index (SII) are emerging as relevant factors in oncology. This study evaluates their associations with established prognostic classifications in prostate cancer (PCa): the Cambridge Prognostic Groups (CPG) and the International Society of Urological Pathology (ISUP) grading system.

**Methods:**

A retrospective cohort study was conducted on 272 prostate cancer patients using systematically collected clinical data. We calculated and analyzed NLR, PLR, and SII concerning CPG and ISUP scores.

**Results:**

Statistically significant relationships were found between NLR, SII, and prognostic classifications. PLR did not demonstrate any significant association.

**Conclusion:**

NLR and SII may be helpful to prognostic factors in PCa, particularly in outpatient settings. However, further multi-center validation and establishing standardized cut-off values are necessary.

## Introduction

Prostate cancer (PCa) is the most commonly diagnosed cancer among men on a global scale, ranking as the fifth leading cause of cancer-related deaths among men, with 1,414,249 new cases and 375,000 deaths reported globally in 2020 (1, 2). The initial steps in diagnosing PCa include testing prostate-specific antigen (PSA) levels and conducting a digital rectal examination (DRE). If either of these evaluations suggests abnormal results, advanced imaging such as multiparametric MRI is used to examine the prostate further, followed by a prostate biopsy and Gleason scoring to determine cancer aggressiveness (3). These assessments help classify patients into risk groups, guiding appropriate treatment strategies. However, aside from PSA monitoring and DRE, few diagnostic tools are readily available in outpatient settings.

Recent research has emphasized the role of systemic inflammation in the development and progression of various cancers, including PCa. For instance, elevated levels of inflammation have been associated with a higher risk of adverse pathological characteristics and biochemical recurrence following surgery (4, 5).

Inflammatory markers derived from routine blood tests, such as the neutrophil-to-lymphocyte ratio (NLR), systemic immune-inflammation index (SII), and platelet-to-lymphocyte ratio (PLR), have been linked to treatment outcomes, pathological features, and biochemical recurrence in PCa (6–12). NLR is a well-studied marker that assesses systemic inflammation and is derived from the ratio of neutrophils to lymphocytes. Elevated NLR values have been associated with more aggressive tumor behavior and poorer prognosis in PCa and other malignancies (6, 13). SII is a marker that integrates platelet, neutrophil, and lymphocyte counts, reflecting both inflammatory and immune responses in cancer patients. Higher values have been consistently associated with worse prognosis across multiple cancer types, including prostate cancer (14). PLR, calculated by dividing the platelet count by the lymphocyte count, has been associated with worse survival rates in various malignancies, including PCa. Meta-analyses have demonstrated that elevated PLR correlates with poor disease-free survival (DFS) and overall survival (OS) in PCa (15).

Given their availability in routine blood tests, these inflammatory markers offer a cost-effective, noninvasive and easily accessible addition to existing diagnostic tools, potentially enhancing PCa evaluation in outpatient settings.

This study explores the association between inflammatory markers (NLR, PLR, SII) and prostate cancer (PCa) risk groups as classified by Cambridge Prognostic Groups and the International Society of Urological Pathology (ISUP) grade groups. The Cambridge Prognostic Groups, introduced in 2016, represent a more refined 5-tier model incorporating ISUP grade group, PSA levels, and clinical T-stage (cT-stage), demonstrating significantly more precise risk stratification compared to the present 3-tier European Association of Urology (EAU) classification (16, 17).

It is hypothesized that specific inflammatory markers could act as early and accessible disease severity or progression indicators, potentially enhancing early detection and patient stratification in clinical practice. Despite existing research, there is no clear consensus on the prognostic or predictive value of these inflammatory markers in PCa. Notably, this study is the first to evaluate the relationship between inflammatory markers and PCa risk groups, specifically within the context of the Cambridge Prognostic Groups, highlighting its novel contribution to the field.

Our research aims to address the current gap in prostate cancer diagnostics, where tools for accurate early detection and risk stratification remain limited. By incorporating easily measurable inflammatory markers into clinical practice, this study could pave the way for more cost-effective, noninvasive diagnostic approaches that complement existing methods. Establishing the clinical utility of these inflammatory markers could lead to their integration into routine screenings, allowing for better management of PCa patients and potentially reducing the burden of advanced disease.

## Materials and methods

### Study design

This retrospective cohort study, based on systematically collected clinical data, aims to investigate the predictive value of inflammatory markers, specifically the NLR, PLR, and SII, in assessing the risk of malignancy and disease progression in PCa patients. The study population consists of individuals diagnosed with PCa who have undergone diagnostic evaluations and staging procedures.

Patient data were categorized based on the CPG and the ISUP grading system. Our preliminary analysis showed no statistically significant differences within adjacent subgroups, which led us to condense the original classifications. The CPG scale was dichotomized into Groups 1–2 versus 3–5, reflecting current treatment paradigms, where CPG 1–2 patients are often managed conservatively, and CPG 3–5 patients more commonly receive definitive treatment. The ISUP grades were similarly grouped into ISUP 1–2 versus 3–5, in line with standard clinical thresholds separating low- and intermediate-grade disease from high-grade malignancies. This approach improved statistical power and aligned with clinically relevant prostate cancer risk stratification thresholds.

### Participants and data collection

The study population consisted of 272 patients diagnosed with prostate cancer (PCa) who underwent diagnostic evaluations followed by radical prostatectomy (RP) at the University Centre of Excellence in Urology, Wroclaw Medical University, Poland, between 2017 and 2022. Inclusion criteria included histologically confirmed PCa through biopsy, a complete blood count with differential, clinical staging, and surgical intervention via either laparoscopic radical prostatectomy (LRP) or open radical prostatectomy. Patients with positive lymph node involvement were also included in the study. Exclusion criteria encompassed individuals with metastatic PCa, those who received neoadjuvant therapy, and patients with incomplete blood count data.

Data were collected from medical records from August 2017 to October 2022. This collection includes demographic information, laboratory results, and histopathological findings. Specifically, the inflammatory markers NLR, PLR, and SII were derived from the complete blood count and platelet count obtained prior to treatment.

### Group classification

Patients were classified according to the Cambridge prognostic group, which categorizes PCa based on histological features and clinical parameters. The data were further analyzed by consolidating the five Cambridge prognostic groups into two categories: C1-C2 (C12) and C3-C5 (C345) to evaluate statistical differences. The same consolidation approach was applied to the ISUP grading system I1-I2 (I12) and I3-I5 (I345).

### Statistical analysis

Descriptive statistics were used to summarize the characteristics of the patient cohort. Correlation analyses were conducted to assess the relationships between the Neutrophil-to-Lymphocyte Ratio (NLR), Systemic Immune-Inflammation Index (SII), and Platelet-to-Lymphocyte Ratio (PLR) in relation to both the Cambridge Prognostic Groups (CPG) and ISUP grade groups. The Shapiro-Wilk test was employed to assess normality hypotheses. Comparative analyses within groups were performed using the Mann-Whitney U-test, with statistical significance set at p < 0.05. Additionally, Receiver Operating Characteristic (ROC) curves were plotted for each predictor (NLR, SII, and PLR), and the area under each curve (AUC) was calculated to evaluate predictive accuracy. Because this was an exploratory, hypothesis-generating analysis, we did not apply a formal correction for multiple testing (e.g., Bonferroni). This decision aimed to minimize the risk of overlooking potentially relevant associations due to increased Type II errors. All results should be interpreted cautiously in this context. All calculations were carried out using GraphPad version 10.3.1.

## Results

### Patient population

A total of 272 patients diagnosed with PCa were included in the study. The demographic characteristics of the patient population are summarized in **Table 1**.

**Table 1:** The demographic characteristics of the patient population.

	Entire cohort	Cambridge 1 (C1)	C2	C3	C4	C5	C12	C345
Group size (%)	272 (100)	58 (21.3)	68 (25)	49 (18)	55 (20.2)	42 (15.4)	126 (46.3)	146 (53.7)
Age median (range) [years]	66 (46-78)	64.5 (4675)	65 (49-74)	68 (51-78)	66 (47-73)	65 (52-77)	65 (46-75)	67 (47-78)
BMI median (±SD)	27.8 (4.1)	27.6 (3.8)	29.1 (4.5)	26.4 (4)	28 (4.2)	28.4 (4.2)	28.1 (4.2)	27.7 (4)
Preoperative PSA [ng/ml] median (IQR)	9.3 (9.9)	6.1 (2.5)	9.7 (8.3)	10.5 (7.6)	11.6 (13.2)	20.5 (24.5)	7 (4.8)	12 (15.1)
pT2 n (%)	142 (52.2)	47 (81)	42 (61.8)	27 (55.1)	22 (40)	4 (9.5)	89 (70.6)	53 (36.3)
pT3 n (%)	130 (47.8)	11(19)	26 (38.2)	22 (44.9)	33 (60)	38 (90.5)	37 (29.4)	93 (63.7)
ISUP 1 quantity (%)	23(8.5)	15 (25.9)	7 (10.3)	1 (2)			22 (17.5)	1 (0.7)
ISUP 2 quantity (%)	105 (38.6)	33 (56.9)	30 (44.1)	22 (44.9)	20 (36.4)		63 (50)	42 (28.8)
ISUP 3 quantity (%)	95 (34.9)	7 (12.1)	223 (33.8)	23 (46.9)	25 (45.5)	17 (40.5)	30 (23.8)	65 (44.5)
ISUP 4 quantity (%)	20 (7.35)	2 (3.45)	2 (2.9)	1 (2)	8 (14.5)	7 (16.7)	4 (3.2)	16 (11)
ISUP 5 quantity (%)	29 (10.7)	1 (1.7)	6 (8.8)	2 (4.1)	2 (3.6)	18 (42.9)	7 (5.6)	22 (15.1)
pN0 quantity (%)	227 (83.5)	58 (100)	65 (95.6)	41 (83.7)	44 (80)	33 (78.6)	123 (97.6)	118 (80.8)
pN1 quantity (%)	45 (16.5)	0	3 (4.4)	8 (16.3)	11 (20)	9 (21.4)	3 (2.4)	28 (19.2)

Regarding clinical and histopathological characteristics, the distribution of patients according to the Cambridge classification was as follows: C1: 58 patients, C2: 68 patients, C3: 49 patients, C4: 55 patients, and C5: 42 patients. The distribution of the consolidated Cambridge prognostic groups categories was as follows: C12: 46.32% and C345: 53.68%.

In terms of ISUP grading, the cohort was divided into the following groups: ISUP 1: 23 patients, ISUP 2: 105 patients, ISUP 3: 95 patients, ISUP 4: 20 patients, and ISUP 5: 29 patients. The ISUP grading system showed that 47.06% of all patients were classified as ISUP 1-2 and 52.94% ISUP 3-5.

The inflammatory markers NLR, SII, and PLR were calculated for each patient. The median values for these markers were as follows: NLR – 2.5 (0.6-7.6), SII - 593.3 (102.5-1828.9), PLR – 128.8 (7.5-321.4).

### Neutrophil-to-lymphocyte ratio

#### NLR vs Cambridge

Descriptive statistics of NLR data in Cambridge scale order are presented in **Table 2**. Group C3 exhibited the highest mean NLR value, which may indicate a higher degree of inflammatory response compared to other groups.

**Table 2:** Descriptive statistics of NLR.

Classification	Cambridge 1 (C1)	C2	C3	C4	C5	C1–2	C3–5
Number of cases	58	68	49	55	42	126	146
NLR range (mean) [SD]	0.6–4.8 (2.4) [0.9]	0.7–6.6 (2.7) [1.1]	1.6–7.6 (3.1) [1.4]	0.7–5.5 (2.7) [1.1]	1.1–6.8 (3.0) [1.2]	0.6–6.6 (2.5) [1.0]	0.7–7.6 (2.9) [1.2]
PLR range (mean) [SD]	19.0–292.2 (131.6) [58.2]	7.5–231.0 (128.1) [47.1]	67.8–267.8 (142.0) [48.3]	22.8–321.4 (133.9) [53.7]	78.3–248.3 (147.1) [44.1]	7.5–292.2 (129.7) [52.2]	22.8–321.4 (140.4) [49.2]
SII range (mean) [SD]	102.5–1520.0 (574.4) [290.0]	147.0–1331.0 (618.9) [261.5]	248.6–1829.0 (718.0) [372.7]	175.9–1598.0 (646.3) [307.5]	169.1–1531.0 (685.7) [274.8]	102.5–1520.0 (598.8) [274.4]	169.1–1829.0 (681.2) [320.2]

PLR and SII data in Cambridge prognostic group order.

Statistical differences between groups were calculated and summarized in **Supplementary Material 1**. The analysis revealed that there were no statistical differences between groups C1 and C2. Nevertheless, significant differences were found between other groups, notably between C1 and C3 (p = 0.0242), C1 and C4 (p = 0.050), C1 and C5 (p = 0.0067), and between the consolidated groups C12 and C345 (p = 0.0091).

**Figure 1:**
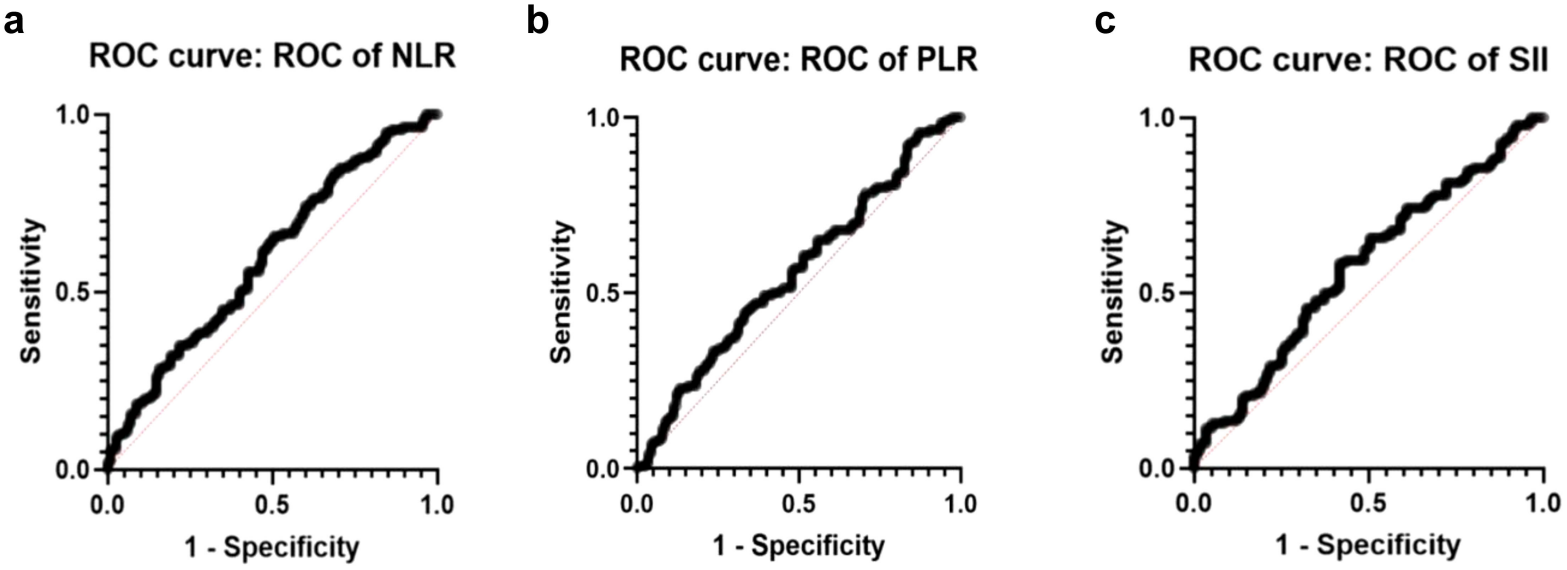
ROC curves of Cambridge prognostic scale. **(a)** ROC of NLR curve (AUC=0.595, std. error: 0.0356 p-value 0.009). **(b)** ROC of PLR curve (AUC=0.5584, std. error: 0.03611 p-value 0.1085). **(c)** ROC of SII curve (AUC=0,5743, std. error: 0,03598 p value 0,0413).

**Figure 1** presents the ROC curve analysis for the grouped data of C12 vs. C345, providing insight into the diagnostic performance of NLR as a predictor of the severity of the disease.

Based on the above findings, it can be concluded that the assumed grouping of data C1 and C2 (cumulated) is reasonable due to the lack of statistically significant differences between groups. It is also worth underlining that NLR differentiates all groups’ configurations based on the C1-reference group. Also, a statistical difference was observed between the cumulative groups C12 and C345.

#### NLR vs ISUP

**Table 3** presents descriptive statistics of NLR values across the ISUP grading groups. Notably, Group I3 exhibited the highest mean NLR value, potentially indicating a greater degree of systemic inflammatory response compared to the other groups. The patient distribution between Groups I1-2 and I3-5 was similar, ensuring balanced representation across lower—and higher-grade categories. Statistical differences between groups are calculated and collected in **Supplementary Material 2**.

**Table 3:** Descriptive statistics of NLR, PLR and SII values across the ISUP scale.

Classification	ISUP1 (I1)	ISUP2 (I2)	ISUP3 (I3)	ISUP4 (I4)	ISUP5 (I5)	ISUP12 (I12)	ISUP345 (I345)
Number of values	22	105	20	96	29	127	145
NLR range (mean) [SD]	0.6–4.4 (2.3) [0.9]	1.1–6.8 (3.2) [1.6]	2.0–7.6 (3.2) [1.3]	0.9–6.2 (2.9) [1.0]	0.6–5.9 (2.5) [1.1]	0.6–5.9 (2.5) [1.0]	0.9–7.6 (3.0) [1.2]
PLR range (mean) [SD]	27.8–281.9 (129.8) [55.6]	69.2–241.5 (143.1) [47.0]	68.5–267.8 (143.5) [48.2]	59.2–321.4 (141.1) [46.7]	7.5–292.2 (129.8) [54.3]	7.5–292.2 (129.8) [54.3]	59.2–321.4 (141.8) [46.6]
SII range (mean) [SD]	106.0–1023.0 (558.4) [245.6]	102.5–1700.0 (607.1) [309.0]	176.4–1598.0 (676.1) [286.1]	361.9–1829.0 (732.7) [348.8]	169.1–1531.0 (711.4) [331.1]	102.5–1700.0 (598.7) [298.7]	169.1–1829.0 (691.0) [303.1]

The analysis reveals no statistically significant differences between Group 1 and Group 2. However, significant differences were identified between specific pairings: I1 vs. I3, I1 vs. I4, and I1 vs. I5. Additionally, when NLR values were used as predictive markers in data grouped by ISUP classifications, a significant difference was observed between ISUP 1-2 and ISUP 3-5 groups.

**Figure 2:**
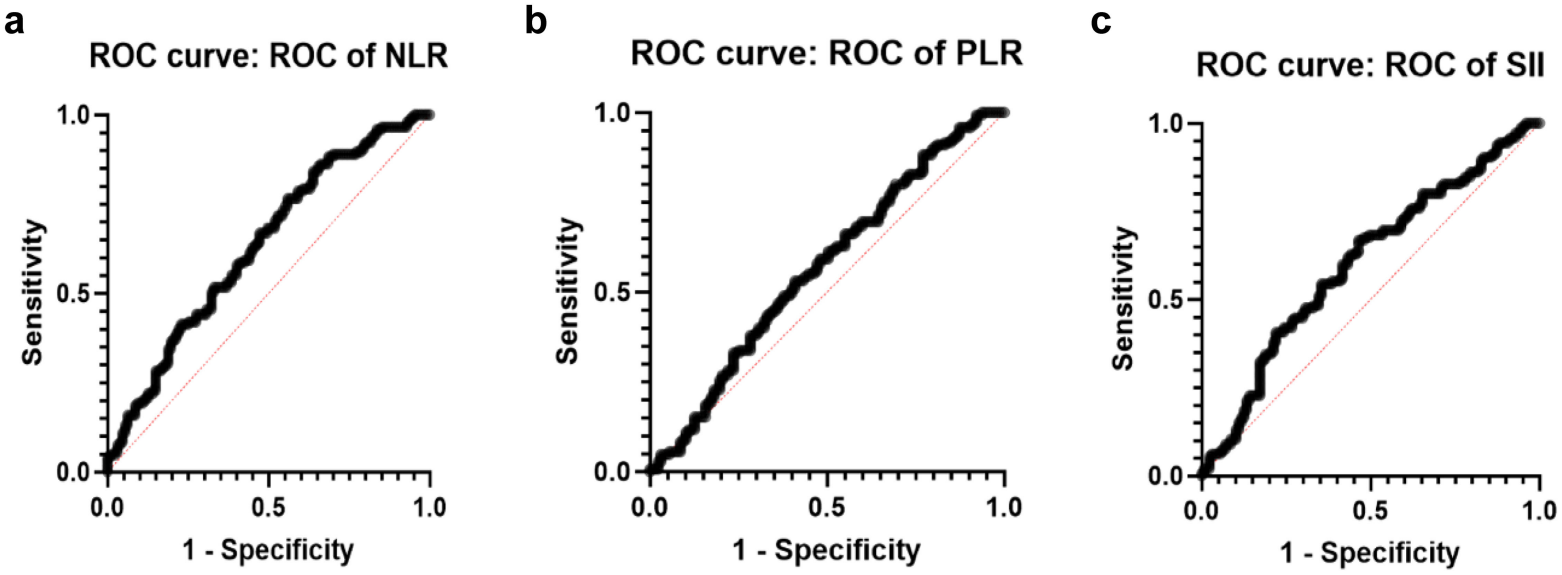
ROC curves on ISUP grading system. **(a)** ROC of NLR curve (AUC=0.6314, std. error: 0.0337 p-value 0.0002). **(b)** ROC of PLR curve for grouped data I12vs.I345 (AUC=0.5663, std. error: 0.03499 p-value 0.0594). **(c)** ROC of SII curve (AUC=0.6030, std. error: 0.03441 p-value 0.0034).

**Figure 2** illustrates the ROC curve for the data categorized into ISUP 1-2 (I12) versus ISUP 3-5 (I345). The ROC analysis evaluates the performance of the predictive approach in distinguishing between lower ISUP grade cases (I12) and higher ISUP grade cases (I345). As can be seen, the ROC curve for NLR demonstrates a notable AUC, indicating the statistically significant value of the predictive approach.

Based on the above, the NLR may be a reliable predictor of outcomes when distinguishing between lower ISUP grade patients (I12) and higher ISUP grade patients (I345), offering valuable prognostic information in this broader classification.

### Platelet-to-lymphocyte ratio

#### PLR vs Cambridge

Descriptive statistics of PLR data in Cambridge scale order are presented in **Table 2**. The standard deviation in C1 was the highest (58.18), indicating a diverse inflammatory response among patients with early-stage disease. At the same time, C5 showed a lower standard deviation (44.07), suggesting more uniformity in inflammatory responses at advanced stages.

According to the data presented in **Supplementary Material 1**, the PLR was ineffective in distinguishing between the groups classified by the Cambridge prognostic categories, as all comparisons yielded p-values greater than the significance threshold of 0.05.

Furthermore, the ROC curve analysis illustrated in **Figure 1** indicates that PLR had a limited capacity to differentiate between the Cambridge prognostic groups in prostate cancer (PCa), performing only slightly better than random chance.

#### PLR vs ISUP

The statistical data for PLR, categorized by ISUP grading groups, is displayed in **Table 3**. It is worth noting that ISUP 1 and ISUP 5 demonstrated identical average PLR values, along with similar standard deviations, indicating comparable variability within these groups. Moreover, the ISUP 3 category exhibited the highest PLR values, suggesting distinct inflammatory or platelet-lymphocyte dynamics within this intermediate-grade classification.

As illustrated in **Supplementary Material 2**, no statistically significant differences were observed among the groups. Furthermore, the ROC curve depicted in **Figure 2** demonstrates that the PLR value lacks a meaningful correlation with the ISUP grading groups.

### Systemic inflammation index

#### SII vs Cambridge

**Table 2** reports the descriptive statistics of PLR data in Cambridge scale order. The highest mean was seen for SII in Cambridge Prognostic Group 3. **Supplementary Material 1** illustrates the calculated statistical differences between groups.

As indicated in **Supplementary Material 1**, the SII parameter can significantly differentiate between the C1 and C5 groups and C12 and C345. These results suggested that SII could serve as a robust biomarker for differential these prognostic groups, which may indicate different levels of systemic inflammatory response and disease severity.

ROC curve analysis of grouped data for C12 vs. C345 shows the diagnostic performance of SII in diagnosing the severity of the disease and its related insight (**Figure 1**). SII correlated well with Cambridge prognostic groups based on both the AUC and p-value, thus confirming its potential role as a prognostic predictor.

#### SII vs ISUP

Descriptive statistics of SII data in ISUP scale order are presented in **Table 3**. The I4 group demonstrated the highest mean value (732.7), unlike the I1 group, which showed the lowest mean value (558.4) accompanied by the most minor standard deviation. That suggests that I4 patients had a more robust inflammatory response, while the measurements in the I1 group were more consistent compared to others.

Statistical comparisons among the groups were conducted and are detailed in **Supplementary Material 2**. Although no significant differences were found within the individual groups, a marked statistical difference is observed between the combined groups I12 and I345.

**Figure 2** illustrates the ROC curve for the grouped data of I12 in comparison to I345, demonstrating statistical significance. That indicates the potential for earlier.

differentiation between lower-grade and higher-grade PCa cases according to the ISUP classification.

## Discussion

The NLR, PLR, and SII are simple clinical markers that indicate a patient’s inflammatory and immune status. Although these ratios have limited predictive value regarding tumor staging (T) and the aggressiveness of PCa, they demonstrate more significant potential for predicting mortality risk and overall survival (OS) in all PCa patients (10, 11, 18, 19). Our research suggests a relationship between these clinical markers’ levels, ISUP GG, and CPGs.

Numerous analyses, including those stratified by ethnicity, PCa subtype, sample size variations, and diverse NLR thresholds, have demonstrated a significant association between OS and NLR levels (18–23). The study by Jiang and Liao highlights the potential of baseline NLR as a significant biomarker in patients with castration-resistant prostate cancer (CRPCa), showing correlations with both progression-free survival (PFS) and OS following docetaxel treatment (21). Similarly, elevated pretreatment NLR has been identified as a negative predictor of response to androgen deprivation therapy (ADT) in patients with metastatic PCa, further supporting its clinical relevance (22). Moreover, Peng et al. underscore the prognostic value of elevated NLR and PLR, identifying them as significant risk factors in patients with metastatic castration-resistant PCa (mCRPC) (23). The restrained stance of Salciccia et al.’s meta-analysis tempers the enthusiasm regarding the predictive utility of NLR and PLR in PCa. While many studies confirm their clinical significance in predicting progression risk and cancer-specific mortality, their effectiveness in assessing advanced disease stages or high-grade PCa aggressiveness remains inconclusive (19). Our study contributes to this ongoing debate by demonstrating statistically significant differences in NLR across cumulative ISUP GGs and CPGs. While ROC curve analyses showed only modest discriminatory ability, these findings suggest that NLR may reflect underlying differences in disease severity. Although unsuitable as a standalone diagnostic tool, NLR could aid risk stratification when interpreted alongside other clinical parameters. Further studies are needed to validate its role and determine its additive value in predictive models.

Pretreatment PLR does show a significant association with OS in patients with localized PCa (24). The meta-analysis conducted by Wang et al. indicated a significant correlation between elevated PLR and inferior disease-free survival (DFS) and OS outcomes in PCa patients (15). Langsenlehner et al. documented that elevated PLR predicts inferior OS among patients with PCa undergoing radiotherapy (25).

Contrary to several previous studies suggesting PLR may serve as a useful prognostic marker, our research shows that PLR cannot effectively distinguish between the groups defined by the ISUP GGs and CPGs in PCa. This result raises important questions about the reliability and applicability of the PLR in risk stratification. Furthermore, our findings support the conclusions of Lee et al. and Murray et al., reinforcing the idea that PLR may not be a dependable prognostic tool in this context (26, 27). The PLR is also found to be an insufficient marker for accurately detecting the presence of PCa (28).

The relationship between SII and prostate cancer risk remains a subject of debate, as some studies suggest that elevated SII may indicate increased risk of PCa but fail to consistently differentiate clinically significant prostate cancer from indolent or benign cases (27, 29, 30). SII is a composite index that integrates neutrophil, platelet, and lymphocyte counts to reflect cancer patients’ systemic inflammatory state and immune balance (30, 31). In contrast, the Gleason scoring system evaluates the histological patterns of prostate cancer based on the architectural features of the tumor (32). In the light of inflammatory markers, SII might complement the Gleason score by providing additional insights into patient prognosis.

The next important issue is ISUP grading system, which refines PCa classification by addressing some of the limitations of the original Gleason scores, providing a more precise risk stratification (33, 34). In our research, higher SII values correlate with more advanced ISUP grades, with significant differences observed between lower (I12) and higher (I345) grade groups. SII also distinguishes between Cambridge Prognostic Groups (e.g., C1 vs. C5, C12 vs. C345), highlighting its potential in assessing systemic inflammation and disease severity. ROC analysis confirms SII’s strong discriminatory capacity, supporting its role as a promising marker for risk stratification and personalized treatment decisions.

An essential thing to take into consideration is investigating the NLR and SII cut-off points, which could provide preliminary indicators for general practitioners and other healthcare professionals. Previous attempts demonstrated significant variability in the reported values across different studies (30, 35). Additional research is needed to determine a reliable cut-off value for practical application in clinical and outpatient settings. Further studies are crucial for guaranteeing precise evaluations of patients and reducing the likelihood of false positives and false negatives.

Our observations suggest that as PCa progresses, inflammatory markers such as NLR become more pronounced and could potentially serve as prognostic indicators in advanced stages of the disease. In line with this, some studies have investigated the predictive effects of PLR, NLR, and SII in prostate cancer, further supporting their potential role in disease progression (36, 37).

Additionally, longitudinal studies tracking the progression of inflammatory markers over time in PCa patients will be crucial for understanding their role in disease evolution and response to treatment, helping to refine risk stratification and personalize treatment plans.

### Limitations and future directions of this study

This study has several limitations that should be acknowledged. Although the clinical data were collected in a systematic and standardized manner, the absence of a predefined prospective study protocol and the retrospective nature of the analysis limits the ability to draw causal inferences. The results may still be influenced by biases, such as confounding or information bias. Moreover, as the investigation was conducted at a single center, the generalizability of the findings may be limited; outcomes observed in this cohort might not fully represent those in broader or more diverse clinical populations, or in institutions with different diagnostic and therapeutic protocols.

The sample size is relatively small for a PCa research, which may affect the statistical robustness and reliability of the findings. Additionally, the inclusion criteria encompassed a broad spectrum of patients, introducing clinical heterogeneity and potentially limiting the interpretability of subgroup analyses. Nevertheless, the study population was drawn from a relatively homogeneous Polish cohort with limited ethnic and socioeconomic diversity, which may help reduce potential confounders and strengthen internal consistency.

Importantly, predefined cut-off values for inflammatory markers were not applied. Instead, continuous variables were analyzed to explore their associations with established prognostic systems. This underlines the preliminary nature of our study, aimed at identifying potential relationship between systemic inflammatory indices and prostate cancer severity. These results provide a foundation for hypothesis generation but require further validation before clinical application.

Future directions should include prospective, longitudinal studies with standardized inflammatory marker measurements and predefined cut-off thresholds.

Existing studies exhibit significant variability in how these markers are defined and applied, highlighting the need to establish standardized thresholds to improve their clinical utility and comparability (23, 30). Exploring the dynamic changes of NLR, PLR, and SII throughout disease progression or in response to treatment may offer additional prognostic insights. Longitudinal monitoring of these markers will be crucial for understanding their role in disease evolution and optimizing personalized treatment strategies.

Moreover, integrating inflammatory markers with imaging data and molecular classifiers may enhance the performance of risk stratification models in prostate cancer. While inflammation is known to influence tumor progression and metastasis, our findings are limited to correlations with nonspecific systemic indices (NLR, PLR, SII). As such, any therapeutic implications, including potential links to immunotherapy or targeted anti-inflammatory strategies, remain speculative and warrant further investigation (38, 39).

These limitations and proposed future directions highlight the need for larger, multi-center, prospective studies with long-term clinical follow-up data to validate and expand the findings. We believe that this study contributes valuable data to the existing literature by from an underrepresented Central European population, providing a unique perspective within the global context of prostate cancer research.

## Conclusions

Systemic inflammatory indices such as NLR and SII, were found to be associated with established prognostic classification systems, including CPGs and ISUP grades, in patients with prostate cancer. These results indicate that NLR and SII may reflect aspects of disease severity captured by existing risk stratification tools. In contrast, PLR did not demonstrate a significant association in this cohort. Further studies, particularly with follow-up and clinical outcome data, are necessary to clarify the prognostic value of inflammatory markers in prostate cancer evaluation.

## Data Availability

The raw data supporting the conclusions of this article will be made available by the authors, without undue reservation.
