# Supplementary material for: Inflammatory-based prognostic indicators in prostate cancer: evaluating NLR, PLR, and SII in relation to Cambridge and ISUP classifications
Source: Front Oncol. 2025 Jul 4;15:1595000. doi: 10.3389/fonc.2025.1595000 (PMC12270877; doi:10.3389/fonc.2025.1595000)
Supplement: Supplementary file 1 [file Table1.docx]

| **Supplementary material 1 (supp mat 1).** Comparison of differences between (NLR, PLR and SII) groups in Cambridge prognostic groups. | | | | | |
| --- | --- | --- | --- | --- | --- |
|  | C1vsC2 | C1vsC3 | C1vsC4 | C1vsC5 | C12vsC345 |
| NLR |  |  |  |  |  |
| p value | 0.1883 | 0.0242 | 0.05 | 0.0067 | 0.0091 |
| PLR |  |  |  |  |  |
| p value | 0.8965 | 0.2713 | 0.734 | 0.067 | 0.1087 |
| SII |  |  |  |  |  |
| p value | 0.2562 | 0.0583 | 0.1471 | 0.0276 | 0.0414 |
| NLR – neutrophil-to-lymphocyte ratio PLR – platelet-to-lymphocyte ratio SII – systemic immune-inflammation index C1–C5 – Cambridge Prognostic Groups C1–2 vs C3–5 – simplified dichotomized grouping reflecting clinical risk stratification  All *p* values were calculated using appropriate non-parametric tests (e.g., Mann–Whitney U test or Kruskal–Wallis test with post hoc analysis) depending on distribution. P < 0.05 was considered statistically significant. | | | | | |
