# Supplementary material for: Inflammatory-based prognostic indicators in prostate cancer: evaluating NLR, PLR, and SII in relation to Cambridge and ISUP classifications
Source: Front Oncol. 2025 Jul 4;15:1595000. doi: 10.3389/fonc.2025.1595000 (PMC12270877; doi:10.3389/fonc.2025.1595000)
Supplement: Supplementary file 2 [file Table2.docx]

| **Supplementary material 2 (supp mat 2).** Comparison of differences between groups (NLR) in the ISUP scale (supp mat) | | | | | |
| --- | --- | --- | --- | --- | --- |
|  | I1vs I 2 | I 1vs I 3 | I 1vs I4 | I 1vs I5 | I12vs I345 |
| NLR |  |  |  |  |  |
| p value | 0.6925 | 0.0275 | 0.0150 | 0.0625 | 0.0002 |
| PLR |  |  |  |  |  |
| p value | 0.9076 | 0.4038 | 0.3487 | 0.3096 | 0.0596 |
| SII |  |  |  |  |  |
| p value | 0.8719 | 0.1526 | 0.1286 | 0.0744 | 0.034 |
| NLR – neutrophil-to-lymphocyte ratio PLR – platelet-to-lymphocyte ratio SII – systemic immune-inflammation index I1–I5 – ISUP Grade Groups I1–2 vs I3–5 – dichotomized grouping reflecting clinical risk (low vs intermediate/high grade)  All *p* values were calculated using non-parametric statistical tests (e.g., Mann–Whitney U test or Kruskal–Wallis test with post hoc analysis). P < 0.05 was considered statistically significant. | | | | | |
